# Supplementary material for: HIVprotI: an integrated web based platform for prediction and design of HIV proteins inhibitors
Source: J Cheminform. 2018 Mar 9;10:12. doi: 10.1186/s13321-018-0266-y (PMC5845081; doi:10.1186/s13321-018-0266-y)
Supplement: Supplementary file 2 — Additional file 2. Source code of HIVProtI web server. [file 13321_2018_266_MOESM2_ESM.zip › HIVprotI_Source-code/hivprotI/weka-3-6-6/doc/weka/filters/unsupervised/attribute/ChangeDateFormat.html]

ChangeDateFormat


---


|  |  |  |
| --- | --- | --- |
| **Overview** \| **Package** \| **Class** \| **Tree** \| **Deprecated** \| **Index** \| **Help** \| **Weka's home** | |  |
| **PREV CLASS**   **NEXT CLASS** | **FRAMES**    **NO FRAMES**     **All Classes** |  |
| SUMMARY: NESTED \| FIELD \| CONSTR \| METHOD | DETAIL: FIELD \| CONSTR \| METHOD |  |


---


## weka.filters.unsupervised.attribute Class ChangeDateFormat

```
java.lang.Object
  weka.filters.Filter
      weka.filters.unsupervised.attribute.ChangeDateFormat
```

**All Implemented Interfaces:**: java.io.Serializable, CapabilitiesHandler, OptionHandler, RevisionHandler, StreamableFilter, UnsupervisedFilter

---

``` public class ChangeDateFormat extends Filter implements UnsupervisedFilter, StreamableFilter, OptionHandler ```

Changes the date format used by a date attribute. This is most useful for converting to a format with less precision, for example, from an absolute date to day of year, etc. This changes the format string, and changes the date values to those that would be parsed by the new format.


Valid options are:

```
 -C <col>
  Sets the attribute index (default last).
```

```
 -F <value index>
  Sets the output date format string (default corresponds to ISO-8601).
```

**Version:**
:   $Revision: 5543 $

**Author:**
:   Len Trigg

**See Also:**: Serialized Form

---

**Constructor Summary**

`ChangeDateFormat()`


| **Method Summary** | |
| --- | --- |
| `java.lang.String` | `attributeIndexTipText()` |
| `java.lang.String` | `dateFormatTipText()` |
| `java.lang.String` | `getAttributeIndex()`             Gets the index of the attribute converted. |
| `Capabilities` | `getCapabilities()`             Returns the Capabilities of this filter. |
| `java.text.SimpleDateFormat` | `getDateFormat()`             Get the date format used in output. |
| `java.lang.String[]` | `getOptions()`             Gets the current settings of the filter. |
| `java.lang.String` | `getRevision()`             Returns the revision string. |
| `java.lang.String` | `globalInfo()`             Returns a string describing this filter |
| `boolean` | `input(Instance instance)`             Input an instance for filtering. |
| `java.util.Enumeration` | `listOptions()`             Returns an enumeration describing the available options |
| `static void` | `main(java.lang.String[] argv)`             Main method for testing this class. |
| `void` | `setAttributeIndex(java.lang.String attIndex)`             Sets the index of the attribute used. |
| `void` | `setDateFormat(java.text.SimpleDateFormat dateFormat)`             Sets the output date format. |
| `void` | `setDateFormat(java.lang.String dateFormat)`             Sets the output date format. |
| `boolean` | `setInputFormat(Instances instanceInfo)`             Sets the format of the input instances. |
| `void` | `setOptions(java.lang.String[] options)`             Parses a given list of options. |

**Methods inherited from class weka.filters.Filter**

`batchFilterFile, batchFinished, filterFile, getCapabilities, getOutputFormat, isFirstBatchDone, isNewBatch, isOutputFormatDefined, makeCopies, makeCopy, numPendingOutput, output, outputPeek, toString, useFilter, wekaStaticWrapper`

**Methods inherited from class java.lang.Object**

`equals, getClass, hashCode, notify, notifyAll, wait, wait, wait`

**Constructor Detail**

### ChangeDateFormat

```
public ChangeDateFormat()
```


**Method Detail**

### globalInfo

```
public java.lang.String globalInfo()
```

:   Returns a string describing this filter

    :   **Returns:**: a description of the filter suitable for displaying in the explorer/experimenter gui

---


### getCapabilities

```
public Capabilities getCapabilities()
```

:   Returns the Capabilities of this filter.

    :   **Specified by:**: `getCapabilities` in interface `CapabilitiesHandler` **Overrides:**: `getCapabilities` in class `Filter`
    :   **Returns:**: the capabilities of this object **See Also:**: `Capabilities`

---


### setInputFormat

```
public boolean setInputFormat(Instances instanceInfo)
                       throws java.lang.Exception
```

:   Sets the format of the input instances.

    :   **Overrides:**: `setInputFormat` in class `Filter`
    :   **Parameters:**: `instanceInfo` - an Instances object containing the input instance structure (any instances contained in the object are ignored - only the structure is required). **Returns:**: true if the outputFormat may be collected immediately **Throws:**: `java.lang.Exception` - if the input format can't be set successfully

---


### input

```
public boolean input(Instance instance)
              throws java.lang.Exception
```

:   Input an instance for filtering.

    :   **Overrides:**: `input` in class `Filter`
    :   **Parameters:**: `instance` - the input instance **Returns:**: true if the filtered instance may now be collected with output(). **Throws:**: `java.lang.Exception` - if the input format was not set or the date format cannot be parsed

---


### listOptions

```
public java.util.Enumeration listOptions()
```

:   Returns an enumeration describing the available options

    :   **Specified by:**: `listOptions` in interface `OptionHandler`
    :   **Returns:**: an enumeration of all the available options

---


### setOptions

```
public void setOptions(java.lang.String[] options)
                throws java.lang.Exception
```

:   Parses a given list of options.


    Valid options are:

    ```
     -C <col>
      Sets the attribute index (default last).
    ```

    ```
     -F <value index>
      Sets the output date format string (default corresponds to ISO-8601).
    ```

    :   **Specified by:**: `setOptions` in interface `OptionHandler`
    :   **Parameters:**: `options` - the list of options as an array of strings **Throws:**: `java.lang.Exception` - if an option is not supported

---


### getOptions

```
public java.lang.String[] getOptions()
```

:   Gets the current settings of the filter.

    :   **Specified by:**: `getOptions` in interface `OptionHandler`
    :   **Returns:**: an array of strings suitable for passing to setOptions

---


### attributeIndexTipText

```
public java.lang.String attributeIndexTipText()
```

:   **Returns:**: tip text for this property suitable for displaying in the explorer/experimenter gui

---


### getAttributeIndex

```
public java.lang.String getAttributeIndex()
```

:   Gets the index of the attribute converted.

    :   **Returns:**: the index of the attribute

---


### setAttributeIndex

```
public void setAttributeIndex(java.lang.String attIndex)
```

:   Sets the index of the attribute used.

    :   **Parameters:**: `attIndex` - the index of the attribute

---


### dateFormatTipText

```
public java.lang.String dateFormatTipText()
```

:   **Returns:**: tip text for this property suitable for displaying in the explorer/experimenter gui

---


### getDateFormat

```
public java.text.SimpleDateFormat getDateFormat()
```

:   Get the date format used in output.

    :   **Returns:**: the output date format.

---


### setDateFormat

```
public void setDateFormat(java.lang.String dateFormat)
```

:   Sets the output date format.

    :   **Parameters:**: `dateFormat` - the output date format.

---


### setDateFormat

```
public void setDateFormat(java.text.SimpleDateFormat dateFormat)
```

:   Sets the output date format.

    :   **Parameters:**: `dateFormat` - the output date format.

---


### getRevision

```
public java.lang.String getRevision()
```

:   Returns the revision string.

    :   **Specified by:**: `getRevision` in interface `RevisionHandler` **Overrides:**: `getRevision` in class `Filter`
    :   **Returns:**: the revision

---


### main

```
public static void main(java.lang.String[] argv)
```

:   Main method for testing this class.

    :   **Parameters:**: `argv` - should contain arguments to the filter: use -h for help


---


|  |  |  |
| --- | --- | --- |
| **Overview** \| **Package** \| **Class** \| **Tree** \| **Deprecated** \| **Index** \| **Help** \| **Weka's home** | |  |
| **PREV CLASS**   **NEXT CLASS** | **FRAMES**    **NO FRAMES**     **All Classes** |  |
| SUMMARY: NESTED \| FIELD \| CONSTR \| METHOD | DETAIL: FIELD \| CONSTR \| METHOD |  |


---
